# Speculations on the Nature of Tubercular Consumption

**Published:** 1853-01

**Authors:** Dinwiddie B. Phillips

**Affiliations:** Assistant Surgeon, U. S. N., Member of Acad. of N. S., Philad.


*Speculations on the Nature of Tubercular Consumption.* By DINWIDDIE B. PHILLIPS, M. D., Assistant Surgeon, U. S. N., Member of Acad. of N. S., Philad.

If in offering for publication the following speculations regarding the nature of Phthisis Pulmonalis, the writer is sufficiently fortunate to engage the attention and invite the aid of abler minds to the investigation of the subject, he will be duly repaid for the time devoted to its examination.

He does not pretend that he has undoubtedly discovered the true pathology of this disease, or an infallible and ever-present cure; but having been induced to adopt his opinions, from what to him seems a preponderance of evidence in their favor, he offers them to the members of the medical profession, to be by them received or rejected, as their wisdom may dictate.

The most which we can learn in relation to the pathology of Phthisis, as treated of by the multitude of medical writers, amounts to but little more than a candid confession of ignorance upon the subject. We are told that it is the result of an abnormal and morbid state of nutrition; that its cause and nature are alike unknown. And when we review the various and conflicting plans recommended for its treatment, and behold the almost innumerable host of heterogenous medical compounds which have been from time to time lauded as the great elixirs of life to the suffering patient, it would seem that any systematic attempt to investigate the nature of the disease, and philosophically oppose its invasions even, although visionary and erroneous, is at least preferable to an entirely lethargic and empirical guesswork, and should therefore claim an indulgent and charitable consideration.

Mr. John Simon, in his work entitled "General Pathology," gives the following description of tubercle:

"Microscopical examination of tubercle shows the following principal ingredients: 1st, a substance which constitutes distinctively the bulk of the gray granulations, and which in its general character is *identical with the matter of condensed fibrinous concretion*; namely, a dense, transparent, and almost homogeneous stroma, soluble in acetic acid and in the alkalies; 2d, granular material often in overwhelming abundance, especially in the yellow tubercle, (where it is superadded to the former constituent) and consisting partly of fibriniform granules, partly molecular oil; 3d, aborted cytoblasts, dark, condensed, mis-shapen, angular, insoluble in acetic acid."

May we not assume, that the sum and substance of the tuberculous condition consists in a precipitation of unduly oxygenated fibrinous elements from the blood, from immediately exciting causes?

Let us see what arguments or facts can be adduced to sustain such an opinion. Mr. Simon, in treating of Cyanosis, uses the following language:

"There is one signal peculiarity which attends this chronic venous condition of the blood, and which I must not leave unmentioned. Not only in extreme cases of Cyanosis, but in all chronic diseases, where, from any causes whatever, there is defective arterialization of the blood, the patient enjoys one privilege. He is exempt (perhaps absolutely, but, at least, *all but absolutely* exempt) from tubercular diseases. And as the circumstances which interfere with due arterialization of the blood are of the most various kinds (some of them acting merely mechanically,) so we are justified in inferring from the exemption just specified, that the condition of the system in which tubercle is deposited is incompatible with venosity of the blood."

Now, there is one peculiarity of venous blood which alone can account for the singular fact stated above. Venous blood is much less prone than arterial to deposit its fibrin. This fact is demonstrated by post-mortem examinations; which always, (or nearly always,) exhibit fibrinous concretions or deposits in the arteries and left side of the heart, whilst the veins and right side are free from them. Mr. Simon also proves the difference in the facility with which arteries and veins give up this element of their composition. He says, (in the work before alluded to,) "I have carried a single thread by means of a very fine needle, transversely through the artery and vein of a dog, leaving it there so that it might cut the stream, and I have done this repeatedly, sometimes in the femoral vessels, sometimes with the carotid and jugular, sometimes with the aorta and cava. I have suffered the thread to remain during a period of from twelve to twenty four-hours. My experiments have given me, as a uniform result, that the arterial blood, with the utmost readiness, deposits its fibrin on the thread; the venous blood with the utmost reluctancy."

Thus, then, the only condition in which the system seems perfectly exempt from the tuberculous deposit, is that in which, from want of proper arterialization, or oxydation, the blood refuses to part with its fibrin, or else does so with greatest difficulty. Again, in noticing the favorite locality of tubercle, we find it originally commencing on the lungs; here the blood is necessarily more highly oxygenated than in any other portion of the body, and here per consequence, its first attack is made; and not only in the lungs, but, by preference, in the superior lobes of the lungs. This arises from the fact that, as a general rule, the upper part of the chest is less frequently exercised than the lower, and the circulation in that particular portion, becoming languid and stagnated, as a matter of course fibrin is there first effused. Comparatively speaking, consumption rarely attacks individuals whose occupations cause them to make free use of the muscles of the upper part of the thorax, whereas, it is of common occurrence with those who pursue sedentary occupations, and whose respiration is carried on chiefly by the diaphragm.

It was formerly charged that tight lacing was a prolific cause of tubercle in females. Dr. J. M. Allen, (Professor of Anatomy in Pennsylvania College of Medicine,) has informed the writer, that he has frequently examined cases where this evil habit had been carried to very great excess; and that although the subjects, previous to death, presented every appearance of a scrofulous diathesis, yet, to his astonishment, instead of finding the lungs filled with tubercle, he has universally found them sound and healthy. He accounts for this upon the principle, that the stricture upon the diaphragmatic portion of the chest, produced a necessarily increased action of the muscles upon the upper portion, and that through this means, the circulation there was rendered more active, and tubercle warded off.

It is hoped that no one will infer that the pernicious and abominable practice of tight lacing is for one moment advocated; far from it, but, as it sometimes happens, that one evil may point out a remedy for another, so in this instance, we are taught that one of the best prophylactics against anticipated consumption consists in moderate, but frequent, exercise of the arms and superior portion of the chest.

Formerly, fibrin was thought to constitute the great pabulum from which the healthy tissues of the body were nourished and repaired; now, however, it is generally conceded to be a retrograde condition of albumen, and of but little use in the economy. To those who still maintain the former opinion, a theory of the kind here advocated will probably appear idle and unsatisfactory, but to others, the views herein presented—defective and imperfect as they are—may, at least, seem to claim some shadow of plausibility.

In speaking of the treatment of Phthisis, it is proposed only to hazard a few conjectures upon the influence of some of the most prominent remedies which have been, and are still, recommended. One of the most favored, and probably most effectual, remedies in use is the Oleum Morrhuæ. This probably acts by furnishing fatty matter to unite with the oxygen of the blood in the generation of animal heat, and thus neutralize any excess; preventing an undue accumulation of super-oxygenated fibrin, and preventing a consumption of muscular and nervous tissue; the iodine and bromine contained in it also acting beneficially, by promoting, softening and absorption of any concretions already formed. The iodide of potassium and nitrate of potassa, with the other alkalies administered, may also do good by rendering the fibrin of the blood more soluble, and less liable to precipitation and coagulation; but in order to produce this effect they should be given in large doses, so that some portion of them may be retained in the circulation, otherwise, they will soon be eliminated and carried off by the kidneys, without exerting any salutary influence. Nutritious and unirritating food, by building up the general system, serve also to lessen the quantity and improve in quality the fibrinous elements of the circulation, and by contributing tone and strength to the economy enable it for a longer time to withstand noxious influences of any and every kind. Removal to a warm climate may act in two ways: 1st, by creating revulsion of excitation from the lungs to the liver; next, by affording less oxygen than would be contained in an equal volume of air respired in a colder atmosphere.

Exercise, as a prophylactic, has been before mentioned; it is also highly efficacious as a remedial agent. It should be of such kind as to call into action the muscles of the superior portion of the thorax and, in fact, all that are in anywise, directly or indirectly, concerned in respiration; and its beneficial influence is much enhanced by making it of such a nature as to be agreeable and pleasant instead of forced and obligatory. Riding on horseback has been most highly extolled by many, its action being probably due to the partial fulfilment of the above recommendation, by using the hands, arms, and shoulders, in the government of the animal, with the veins, and also by improving digestion and increasing the general strength. Cases are on record, where persons, who despairing escape from so hopeless a malady, and perfectly reckless of consequences, have engaged in pursuits of the roughest, and apparently, most injurious nature, and who, after travelling on horseback or on foot, and performing real manual labor, have, to the joyful surprise of their friends, entirely recovered from their dangerous condition, and become both strong and hearty. Sailors, soldiers and others, engaged in occupations of similar physical necessities, although much subject to inflammatory affections of the chest, are in general less prone to tubercle than those whose condition in life would apparently better protect them from its ravages.

There are many remedies prescribed and used of a palliative nature, but it is scarcely necessary to mention them in an article of this kind; the limits of the treatise and time for its preparation, both forbid a more extended examination of the subject. The ideas here advanced have been hastily (perhaps too hastily) prepared and presented; but should they induce investigation, or cast any light, however small, upon a disease as obscure in its nature, as dangerous and fatal in its consequences, the highest hopes of the writer will be more than realized.

